# Hormone Replacement Therapy in Endometrial Cancer Survivors: A Meta-Analysis

**DOI:** 10.3390/jcm10143165

**Published:** 2021-07-18

**Authors:** Ambrogio P. Londero, Nadia Parisi, Alice Tassi, Serena Bertozzi, Angelo Cagnacci

**Affiliations:** 1 Clinic of Obstetrics and Gynecology, DAME, University Hospital of Udine, 33100 Udine, UD, Italy; parisi_nadia@libero.it (N.P.); alicetassi9@gmail.com (A.T.); 2 Ennergi Research (Non-Profit Organisation), 33050 Lestizza, UD, Italy; dr.bertozzi@gmail.com; 3 Breast Unit, University Hospital of Udine, 33100 Udine, UD, Italy; 4 Clinic of Obstetrics and Gynecology, DINOGMI, IRCCS San Martino Hospital, University of Genova, 16122 Genova, GE, Italy

**Keywords:** endometrial cancer, hormone replacement therapy, hormone therapy, ethnic origin, recurrence, disease free survival, overall survival, estrogens, progestins

## Abstract

The purpose of this study is to investigate the effect of hormone therapy (HT) on the oncological outcomes of endometrial cancer (EC) survivors. A systematic literature review was conducted in July 2021 to identify studies detailing the effect size for the relationship between HT use in EC and oncological outcomes (survival and disease recurrence). This included studies that evaluated the different recurrence rates among women treated for EC who subsequently underwent HT and those who did not. The collected studies were evaluated for quality, heterogeneity, and publication bias, and a pooled odds ratio (OR) or hazard ratio (HR) was calculated with a confidence interval of 95% (95% CI). In total, 5291 studies were collated, and after the review process, one randomized trial and seven observational studies were included, comprising 1801 EC survivors treated with HT and 6015 controls. The time-dependent analysis could be conducted for four studies, and considering the disease-free survival, the pooled HR of 0.90 (95% CI 0.28 to 2.87) showed no significant differences. However, among Black American women treated with continuous estrogen HT, the HR was 7.58 (95% CI 1.96 to 29.31), showing a significantly increased risk of recurrence for women in this ethnic group. Considering the pooled OR of all included studies 0.63 (95% CI 0.48 to 0.83), a significantly reduced risk of recurrence was found among EC survivors treated with HT. Considering the type of HT, the most risk-reducing was combined estrogen and progestin therapy and the cyclic regimen. Although supporting evidence is based mainly upon observational studies, evidence of no increased risk or even decreased risk was generally found, apart from in Black American women where a significantly increased recurrence risk was evident. The data are rather reassuring for the short-term administration of HT to symptomatic EC survivors. Future studies with a longer follow-up are necessary to better clarify the long-term effects of HT.

## 1. Introduction

In developed countries, endometrial cancer (EC) is the most common gynecological malignancy, with cases expected to increase further in the near future [1].

Surgery is the main treatment and staging procedure for early EC stages. This consists mainly of hysterectomy and bilateral salpingo-oophorectomy, leading to a favorable prognosis for stage I tumors [1,2]. Almost 25% of women are pre-menopausal, despite EC generally being a post-menopausal condition [3], with 4% of EC patients being under 40 years of age [2]. However, menopausal symptoms are commonly reported by pre-menopausal women treated for EC. Compounding this, surgically provoked menopause presents more severe symptoms than spontaneous menopause [4]. Therefore, there is a strong desire to treat these symptoms for women previously treated for EC to permit a significantly improved quality of life.

Although there is no scientific evidence to confirm a harmful effect of hormonal therapy (HT) in women treated for EC, the estrogen-dependent carcinogenesis in EC makes doctors unwilling to prescribe such treatments for these patients [5,6]. A recent meta-analysis concluded that HT had no impact on whether or not EC recurred, but no time-to-event analysis was performed [6]. It is consequently difficult for women treated for EC and their doctors to weigh up the benefits and risks of HT. In this respect, the main aim of this study was to estimate the time-dependent effects of HT on EC survivors with respect to disease-free survival, overall survival, and recurrences via a review of observational and randomized studies. In addition, the secondary aims were to investigate the effect of HT on cancer recurrences in EC survivors where a time-to-event analysis was not feasible, as well as to evaluate the possible confounding factors.

## 2. Materials and Methods

### 2.1. Literature Search Strategy for Review

A literature search was independently performed by three authors (NP, AT, and APL) using a standardized approach, and a systematic review was undertaken using appropriate guidelines [7,8]. Relevant information was gathered from PubMed/Medline, Scopus, Cochrane Central Register for Controlled Trials, Cochrane Central Register of Reviews, EMBASE, Web of Science, Clinicaltrials.gov, and International Clinical Trials Registry Platform (ICTRP) for studies published up to July 2021. Search terms applied were: ’hormone replacement therapy OR hormone therapy OR estrogen replacement therapy OR menopausal hormone replacement therapy OR menopausal estrogen and estrogen-progestin replacement therapy OR HRT OR HT OR ERT OR MHRT OR MRT’ and ’endometrial OR endometrium’ and ’neoplasm OR carcinoma OR cancer’ (query details are presented in Table S1). Meta-information, titles, and abstracts resulting from these queries were examined to exclude any clearly unrelated articles. Following this, full texts of the remaining papers were assessed independently by three authors (NP, AT, and APL) to determine their relevance. Finally, references cited in previous review articles and full articles were used to identify any other relevant articles.

### 2.2. Inclusion and Exclusion Criteria

Papers were included if they satisfied the following inclusion criteria. Eligible study designs were defined as randomized controlled clinical trials, prospective/retrospective cohort studies, nested case-control studies, or population-based case-control studies. Studies were then restricted to those considering patients who received surgical treatment for their EC and those considering HT exposure as an intervention of interest. Eligible studies also had to show data (Kaplan–Meier curves, hazard ratio (HR), odds ratio (OR), relative risk (RR), or adequate data to calculate them) about one of the following outcomes: endometrial cancer recurrence, disease-free survival, or overall survival. Only articles available in the full text regarding human subjects and written in English, Italian, German, French, or Spanish were retained for the analysis. Authors were not contacted for missing data or full text versions. Information from the full text articles reporting geographic locations, the time frame for patient treatment, and type of treatment were noted in order to avoid any potential overlap of the populations. Where there were multiple studies relating to the same set of women treated for EC or presenting potentially overlapping data, the study with better quality or more detailed data was retained, or both reports were retained together if they described different aspects of the same study. Any assessment differences among the reviewers were resolved by a joint re-evaluation of the original article to address them. Articles were excluded if they were written in a language other than English, Italian, German, French, or Spanish, if they were single-arm studies without a control group, or if they were dealing with nonhuman subjects. Furthermore, conference abstracts, case reports, reviews, editorials, or letters to the editor were excluded due to the lack of detail regarding study design and outcome data.

### 2.3. Data Extraction

Using a data extraction form, three independent reviewers (NP, AT, APL) extracted predefined data from the eligible studies encompassing the following information: study design, year of publication, authors, geographical location, population characteristics (age, tumor stage, grade, etc.), number of patients, exposure to HT use, type of HT, duration of HT, duration of follow-up after HT administration, EC recurrences, deaths, HR with a 95% confidence interval (95% CI), or HRs extracted from Kaplan–Meier curves. It was assumed that, when not directly reported, the follow-up after HT initiation was equal to the duration of HT. The HR value was estimated from published reports using methods previously described [9]. If a multivariate analysis was available in the considered study, the adjusted HR with the relative CI was used in our analysis. Moreover, the authors systematically reviewed all included studies for characteristics that could introduce possible bias (e.g., procedures for participant identification, differences in the duration of follow-ups between cases and controls, the comparability of risk factors for recurrence present at diagnosis for cases and controls). Continuous data were collected as mean and standard deviation according to the previously described methods [10,11]. As previously described, any discrepancies in data extraction or the suitability or otherwise of a study were jointly discussed until a consensus was reached [12].

### 2.4. Quality Assessment

The quality of the included studies was independently assessed by NP and AT using the Newcastle–Ottawa Scale as previously described [12], which enables assessment of selection, comparability, and outcomes (cohort studies) or exposure (case-control studies). For the purpose of this study, the following definition was adopted: high-quality studies were those scoring 9 or 8 points on the Newcastle–Ottawa Scale, medium-quality studies were those scoring 7 or 6 points, and low-quality studies were those with a score of <6 points [12]. Discrepancies in the quality assessment were solved as previously described after the discussion, and re-evaluation with a third author (APL) [12,13,14].

### 2.5. Data Analysis

R (version 3.6.2; R Core Team—2019. R: A language and environment for statistical computing. R Foundation for Statistical Computing, Vienna, Austria—URL https://www.R-project.org/) was used for all statistical tests, considering, in this meta-analysis, the *p*-value < 0.05 as significant. A summary statistic considering the HR for survival analysis or OR was calculated. The fail-safe N test, Begg–Mazumdar rank correlation test, and rank correlation test (Egger’s test) of funnel plot asymmetry were used to test publication bias [15,16,17,18]. The between-study heterogeneity was evaluated by the I-square index and the Cochran Q. An I-square index value >50% and a Q statistic *p*-value < 0.10, as previously described, were considered statistically remarkable signs of heterogeneity [19]. Where pertinent, the fixed- or the random-effect model was used to calculate the pooled estimate. The primary outcome was described as HR (with 95% CI) of disease-free survival in EC survivors exposed or not to HT. The ORs (with 95% CI) were also presented for the recurrence rate of EC survivors exposed or not to HT. A priori planned subgroup analysis was, subsequently, performed for the type of HT (considering estrogen-only versus combined HT or cyclic vs. continuous), the tumor staging (stage I, stage I-II, or stage I-III), and the type of study (randomized control trial (RCT), retrospective case-control, or cohort studies). A sensitivity analysis was performed to check the robustness of the pooled effects by removing each study, one by one. This meta-analysis was developed by following the Meta-analysis Of Observational Studies in Epidemiology (MOOSE) guidelines for accurately performing meta-analysis of observational studies [8] and the Preferred Reporting Items for Systematic Reviews and Meta-Analyses (PRISMA) guidelines checklist [7]. The present study is exempt from ethical approval, since this meta-analysis involves only already published anonymous data.

## 3. Results

### 3.1. Search Results

**Figure 1:**
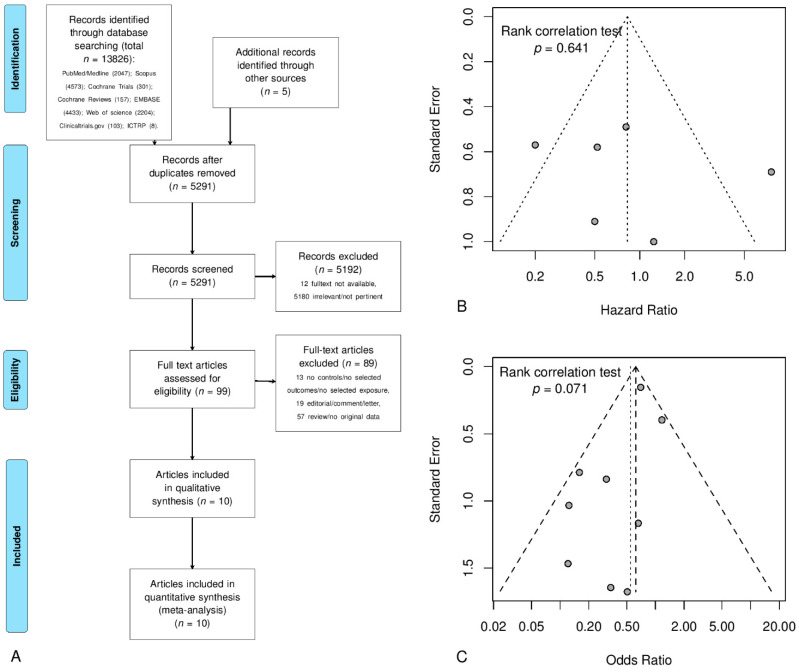
Panel (**A**) Flowchart of study selection process according to PRISMA guidelines. Panel (**B**) Funnel plot of hazard ratio (HR) meta-analysis for disease-free survival/recurrence risk (Rank correlation test *p*-value = 0.641). Panel (**C**) Funnel plot of odds ratio (OR) meta-analysis for recurrence risk (Rank correlation test *p*-value = 0.071).

The flowchart in Figure 1A shows how relevant studies were identified through a search of existing literature. In total, 5291 items were found (Figure 1A) and, after reviewing titles and abstracts, 5192 were removed because they were irrelevant/not pertinent (N = 5180) or a full text was not available (N = 12). Thus, 99 articles were potentially eligible for this review. However, 13 articles either did not present a control group or the selected outcomes/exposures, 19 were editorials, comments, or letters, and 57 were reviews or publications without original data (List S1). List S1 shows both the excluded studies and the 10 eligible articles describing 9 different studies (Figure 1A) [20,21,22,23,24,25,26,27,28,29].

### 3.2. Characteristics of the Studies

Among the 10 papers published between 1986 and 2021, two reported data from a randomized clinical trial [23,25], five were non-randomized cohort studies, and three were case-control studies (Table 1). These observational studies were mostly retrospective, or retrospective from prospective databases. The total number of EC survivors treated with HT was 1867, and the total number of controls was 6077. A total of 7944 subjects were considered in this meta-analysis, including the whole cohort presented by Suriano and coworkers [26]. The detailed characteristics of the included studies are reported in Table 1 and Table S2.

The mean age of EC survivors treated with HT was 54.39 years (95% CI 48.32 to 60.46), and that of controls was 57.74 years (95% CI 53.64 to 61.85) [20,24,25,26,28,29]. In addition, the mean age difference was −3.31 years (95% CI −6.31 to −0.31), showing a significant lower age among EC survivors treated with HT [20,24,25,26,28,29] (Figure S1A). Whilst the article of Lim and coworkers was not considered in our analysis of age due to potential overlap with Cho and coworkers, it is worth commenting that this article also noted that EC survivors treated with HT were younger than their control counterparts [20,21]. Furthermore, in two studies it was not possible to extract the standard deviation of the patients’ ages [22,27]. In a study by Chapman and coworkers, the age was significantly lower in EC survivors treated with HT than in controls, while in the study by Arteaga-Gomez and coworkers, there were no significant differences [22,27].

The mean follow-up time was 63.55 months (95% CI 55.83 to 71.27) (Figure S1B). In three studies, it was possible to assess the difference between cases and controls, and in these, the follow-up was significantly longer in the control group (MD of 14.40 months, 95% CI 5.36 to 23.44) (Figure S1C) [21,26,28].

In two studies, an estrogen-only therapy was used, in the first as a continuous regimen [23,25] and in the second as a cyclic regimen [29]. In a further two studies, continuous combined HT was used [21,24]. In the remaining five studies, HT was a mixture of a combined, estrogen-only regimen, or tibolone [20,22,26,27,28]. Among the HT treatments, conjugated equine estrogen (CEE) was the most common among the included studies.

The disease-free interval before starting HT was an inclusion criterion for three of the studies [21,23,24,25] and for all patients considered in these, the disease-free interval was <5 months (Table S2). In four of the studies, the mean disease-free interval was 38 months (95% CI 10–65) [26,27,28,29], but excluding one of these (the article of Chapman and coworkers), the mean disease-free interval was 21 months (95% CI 11–31) [26,28,29]. In the last two studies, the disease-free interval was unknown [20,22] (Table S2).

In four studies, the mean HT duration was between 23 and <40 months [20,23,25,27,29], and in another four studies, the mean HT duration was between 40 and 67 months [21,22,24,28], while Suriano and co-workers did not report the mean HT duration (Table S2). The pooled mean HT duration was 43.40 months (95% CI 36.52 to 50.27).

Most of the studies do not show significant differences in stage and grade prevalence between the compared groups [21,22,23,24,25,26,27,29]. In one study, data about grade and stage are not reported [20], while in another study, a high number of high-grade tumors was reported in the control group [28]. However, considering the pooled G3 prevalence in the group receiving HT of non-randomized studies, the proportion was 0.09 (95% CI 0.05 to 0.15), while in the control group, the pooled proportion was 0.12 (95% CI 0.09 to 0.16).

### 3.3. Quality Assessment of the Included Studies

The quality of included studies was mostly low (levels IIB-III-IV, CEBM) [30]; in fact, the majority of the studies were non-randomized studies, and the only randomized clinical study presented a sub-optimal power, and was canceled before reaching the predetermined sample size.

Using the 9-point rating Newcastle–Ottawa scale for quality, the median score of the included studies was 8 (IQR 6–9). Six studies were graded 8 or 9 points according to the Newcastle–Ottawa quality assessment scale (high quality), two studies were graded 6 or 7 (medium quality), and one study, 5 (low quality).

The included studies attempted to manage confounding factors in different ways: firstly by restrictive inclusion/exclusion or matching criteria [24,25,26], and secondly by multivariate analysis [20,23,27] (Table S2).

For the comparability section of the Newcastle–Ottawa scale, five studies were granted two points because age and other factors were controlled by means of restrictive inclusion criteria and/or multivariate analysis [20,23,24,25,26,27]. Meanwhile, four studies were granted one point because stage or other factors were controlled [21,22,28,29].

For the exposure/outcome section, two studies lost points for not accurately presenting the method of investigations and for not exhaustively reporting the loss to follow-up [27,29].

### 3.4. Meta-Analysis

**Figure 2:**
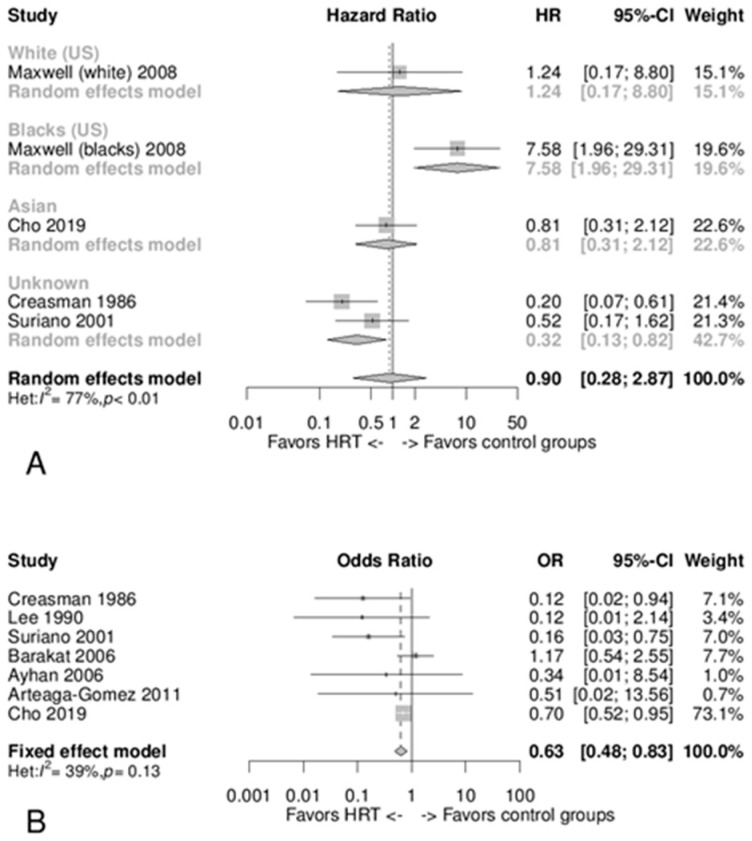
Panel (**A**) Forest plot of hazard ratio (HR) meta-analysis for disease-free survival/recurrence risk showing a pooled random effect model and subgroup analysis by ethnicity (in this analysis, Lim and coworkers were excluded due to a potential overlap with Cho and coworkers). Panel (**B**) Forest plot of odds ratio (OR) meta-analysis for recurrence risk showing a pooled fixed-effect model (in this analysis, Lim and coworkers were excluded due to a potential overlap with Cho and coworkers; and Chapman and coworkers were excluded due to an overlap with Suriano and coworkers).

In Figure 2A, four articles were considered, comprising a total of 7276 patients [20,23,26,29]. For this analysis, the article by Lim and coworkers was excluded due to potential overlap with Cho and coworkers [20,21]. Figure 2A shows the meta-analysis of the studies that present cox regression or Kaplan–Meier data, demonstrating that no significant differences were found between HT users and controls in terms of disease-free survival/risk of EC recurrence considering the pooled HR of 0.90 (95% CI 0.28 to 2.87). Figure 2B shows the meta-analysis of OR values where the pooled result showed a reduced risk of cancer recurrence in patients using HT (OR 0.63 95% CI 0.48 to 0.83). In this analysis, seven articles were considered, incorporating a total of 7548 patients (1692 HT users and 5856 controls) [20,22,24,25,26,28,29]. Here, the study by Lim and coworkers was excluded due to a possible overlap with Cho and coworkers [20,21], whilst the study by Chapman and coworkers was excluded due to an overlap with Suriano and coworkers [26,27]. The effect of individual studies on the pooled effect size was assessed for both previous analyses (Figure 2A,B) with a sensitivity analysis, in which the analysis was repeated, omitting one study at a time, to determine the contribution of each study to the effect size. This sensitivity analysis found no significant differences.

### 3.5. Risk of Bias Assessment

The heterogeneity of the treatments and population characteristics is the main limitation of this analysis. In fact, the eligible studies presented a wide range of variety in HT treatments, ethnicity, follow-up, and tumor stage. Several different subgroup analyses were performed to better interpret the evidence presented in the included studies.

### 3.6. Type of Study Design

**Figure 3:**
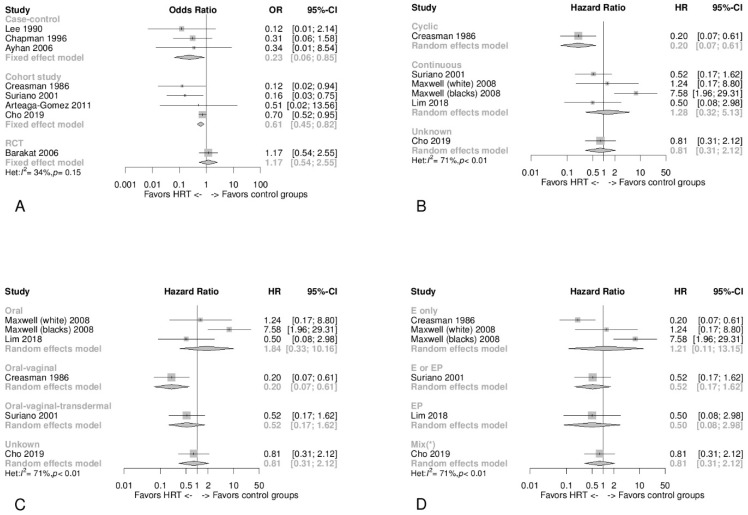
Panel (**A**) Forest plot of odds ratio (OR) meta-analysis for recurrence risk showing a pooled fixed-effect model subgroup analysis by type of study (in this analysis, Lim and coworkers were excluded due to a potential overlap with Cho and coworkers). Panel (**B**) Forest plot of hazard ratio (HR) meta-analysis for disease-free survival/recurrence risk showing a pooled random effect model subgroup analysis by the timing of HT administration (cyclic vs. continuous). Panel (**C**) Forest plot of hazard ratio (HR) meta-analysis for disease-free survival/recurrence risk showing a pooled random effect model subgroup analysis by the method of HT administration. Panel (**D**) Forest plot of hazard ratio (HR) meta-analysis for disease-free survival/recurrence risk showing a pooled random effect model subgroup analysis by type of HT ((*) the mixed subgroup includes patients treated with tibolone).

Firstly, it was only possible to perform an HR meta-analysis in the observational cohort studies and in the randomized clinical study. This assessed the follow-up time, where we observed no significant differences in cancer recurrence between HT users and controls (Figure 2A). Of particular note is the fact that the three cohort studies had a pooled HR of 0.45 (95% CI 0.20 to 1.03) [20,26,29], whilst the two ethnic groups in the Maxwell and coworkers study (White Americans and Black Americans) had a pooled HR of 4.20 (95% CI 0.49 to 35.82) [23]. In Figure 3A, the OR was sub-grouped by study design: only the RCT did not show differences between cases and controls. Cohort and case-control studies showed an advantage of HT in reducing cancer recurrences, with an OR of 0.23 (95% CI 0.06 to 0.85) for the case-control subgroup and an OR of 0.61 (95% CI 0.45 to 0.82) for the cohort studies subgroup.

### 3.7. Ethnicity

An interesting observation emerged from the Maxwell and coworkers study, in that Black American women showed a significantly increased risk of cancer recurrence while undergoing HT [23]. Figure 2A shows that all other ethnic groups considered presented similar HR values, all of which are lower than Black American women. Considering OR stratified by nations the pooled OR of the US was 0.45 (95% CI 0.26 to 0.80) [25,26,28,29], and was similar to Turkey OR 0.34 (95% CI 0.01 to 8.54) [24], to Mexico OR 0.51 (95% CI 0.02 to 13.56) [22], and to Korea OR 0.70 (95% CI 0.52 to 0.95) [20].

### 3.8. Type, Timing, and Duration of HT

**Figure 4:**
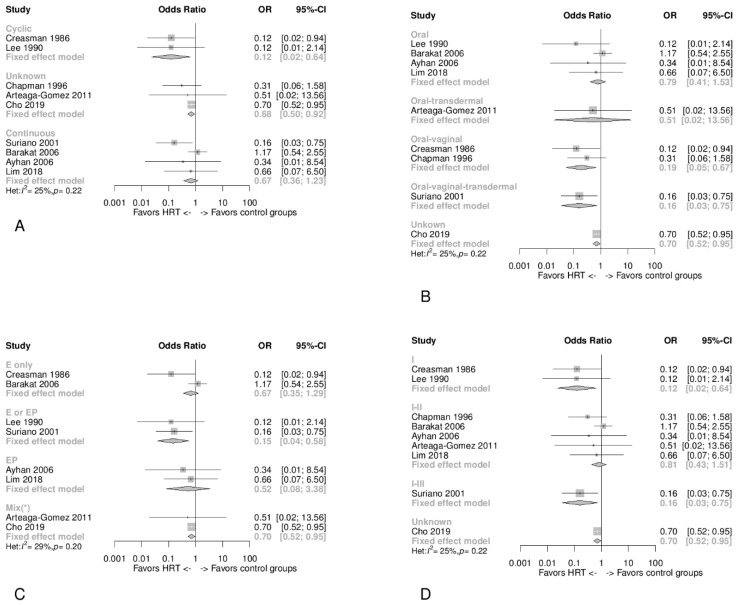
Panel (**A**) Forest plot of odds ratio (OR) meta-analysis for the recurrence risk showing a pooled fixed-effect model subgroup analysis by the timing of HT administration (cyclic vs. continuous). Panel (**B**) Forest plot of odds ratio (OR) meta-analysis for recurrence risk showing a pooled fixed-effect model subgroup analysis by method of HT administration. Panel (**C**) Forest plot of odds ratio (OR) meta-analysis for recurrence risk showing a pooled fixed-effect model subgroup analysis by type of HT (Chapman and coworkers was excluded due to an overlap with Suriano and coworkers; in addition, the mixed subgroup includes patients treated with tibolone). Panel (**D**) Forest plot of odds ratio (OR) meta-analysis for recurrence risk showing a pooled fixed-effect model subgroup analysis by tumor stage.

Figure 3B–D shows the HR for each study and the pooled HR for all HT-type categories. Figure 3C and Figure 4A,B then show the OR for the same HT-type categories. The HR of the cyclic HT regimen showed a significant decrease in EC recurrences (HR 0.20, 95% CI 0.07 to 0.61) (Figure 3B) [29], and the same significant reduction was observed using the OR (pooled OR 0.12, 95% CI 0.02 to 0.64) (Figure 4A) [28,29]. In both Figure 3B and Figure 4A, the pooled HR and OR showed no significant differences for continuous HT regimens. Figure 3C and Figure 4B show that in the subgroups that included a portion of patients where vaginal administration was adopted, a decrease in the risk of recurrence in those undergoing HT was apparent. Figure 3D and Figure 4C show that the subgroups containing the combined regimens (EP) presented a better risk reduction for EC recurrence in HT users.

In Figure S2A,B, the forest plot was stratified by the disease-free interval before HT treatment, showing that the best-performing were the studies with a mean disease-free interval of 21 months (95% CI 11–31). In Figure S2C,D, the mean treatment duration distribution was divided at the median value of 40 months, showing no significant differences for shorter than the median or longer than the median treatment duration in terms of EC recurrence.

### 3.9. Tumor Stage

Figures S3 and S4D display the HR and OR values for each study, respectively, and the pooled values for classes of stage I, stages I–II, stages I–III, and unknown. In Figure 4D, where OR is evaluated, a decrease in recurrence risk was observed in every stratum. In addition, in studies exclusively evaluating stage I EC survivors, HT use had a significant reduction in the risk of EC recurrence considering both OR and HR, respectively, 0.12 (95% CI 0.02 to 0.64) and 0.20 (95% CI 0.07 to 0.61) (Figure 4D and Figure S3) [28,29]. In Figure S3, for the HR categories I–II, I–III, unknown no significant differences are identified.

### 3.10. Publication Bias

Publication bias was examined by means of the funnel plot method, in which the standard error of the effect size of each study was plotted against its effect size (assessed by HR or OR) (Figure 1B,C). No significant publication bias was found, and the rank correlation test of funnel plot asymmetry had a *p*-value of 0.641 for the HR and 0.071 for the OR. In addition, for the OR, the analysis of Begg’s rank correlation test and of the fail-safe N test were performed. Begg’s rank correlation test did not show significant asymmetry of the funnel plot and thus no evidence of publication bias (*p* = 0.910). The fail-safe N test revealed that nine more studies with significant findings are required for the combined two-tailed *p*-value to exceed 0.050.

## 4. Discussion

### 4.1. Key Results

According to this systematic review of published data, HT use had no unfavorable effect on recurrence, disease-free survival, or overall survival after EC treatment. In the studies analyzed, no death attributable to cancer was described in HT or control groups. In addition, this disease-free survival/disease recurrence meta-analysis suggests that HT use by women with a history of EC did not lead to an increase in the risk of EC recurrence (HR 0.90, 95% CI 0.28 to 2.87; and OR 0.63, 95% CI 0.48 to 0.83). This same pattern of results was found in the subgroup analysis by tumor stage, HT type, timing, and duration. However, when analyzing data by ethnicity, Black American women treated with HT were found to be at an increased risk of EC recurrence (HR 7.58, 95% CI 1.96 to 29.31). We also noted that amongst the different types of HT, the most risk-reducing was EP (OR 0.63, 95% CI 0.47–0.84) and the cyclic regimen (OR 0.12, 95% CI 0.02 to 0.64), and considering the tumor stage, HT was most effective in reducing recurrence risk in FIGO I (OR 0.12, 95% CI 0.02–0.64).

### 4.2. Interpretation

A subgroup analysis based on the different variables was conducted to investigate the potential confounding factors across studies. In particular, the subgroup analysis based on study design showed that case-control or cohort studies showed different estimates than RCT. In particular, case controls (OR 0.23, 95% CI 0.06 to 0.85) or cohort studies (OR 0.16, 95% CI 0.45 to 0.82 and HR 0.45, 95% CI 0.20 to 1.03) suggested that HT prevented EC recurrence, whereas those from the RCT (OR 1.17, 95% CI 0.54 to 2.55 and HR 4.20 95% CI 0.49 to 35.82) did not. The difference between non-randomized studies and the randomized clinical trial may be attributable to the design limitations inherent in non-randomized studies, despite the techniques used to remove potential confounding factors. Two confounding factors could be the duration of follow-up and the time elapsed between the end of EC treatment and starting HT. Both these factors could be partially controlled for by a time-dependent analysis—and in fact, the HR of cohort studies does still show protective qualities, but not to the same level of significance as the OR (and there are still different degrees of immortal time bias in the retrospective cohort studies). The immortal time bias happens when participants to treated groups are assigned using information that was observed after time-zero. This bias results in a distortion of observed effects in favor of the treatment under study by conferring a survival advantage to the HT-treated group. The best way to avoid this bias is to align assignment and time-zero as in the RCT [23,25]. Other ways, a time-dependent analysis could partly reduce this bias impact by classifying immortal-time subjects into the unexposed group using person-time definitions. Although these statistical procedures cannot entirely eliminate immortal time bias, none of the included retrospective cohort studies used this type of correction; selection criteria or sub-analysis groups were applied to manage this type of bias (Table S2). In the majority of the eligible studies, the mean disease-free interval before HT was 21 months (95% CI 11–31) [26,28,29], whereas in Chapman and coworkers, it was 87 months [27] (Figure S2B). In addition, most of the EC recurrences occur within the first 24 months of follow-up [31]; therefore, patients that started HT after 24 months were already less likely to experience a recurrence than those starting before (Figure S2B). Another important factor emerging from the non-randomized studies was the difference in patient age between cases and controls (HT users were, on average, 3.31 years younger than controls). Furthermore, a high tumor grade—another important risk-factor for recurrence—was more common in controls than in HT users in the non-randomized studies. All these selection biases could explain the more protective effect of HT that emerged from the non-randomized studies.

Considering that adding progesterone would counter the harmful effects of estrogen on the endometrial tissue, various studies combined progesterone to estrogen treatment [20,24,26,28]. In this subgroup analysis, the combined HT (pooled OR of 0.63, 95% CI 0.47 to 0.84) seems to be slightly more effective than an estrogen-only treatment (OR 0.67, 95% CI 0.35 to 1.29). Furthermore, the pooled HR of combined HT was 0.67 (95% CI 0.32 to 1.40) [20,26], while the pooled HR of estrogen-only pills was 1.21 (95% CI 0.11 to 13.15) [23,25,29]. Two studies used estrogen alone, and showed different results [23,25,29]. The first of these two studies was the randomized controlled study presented in two articles by Barakat and coworkers, and Maxwell and coworkers [23,25]. Barakat and coworkers used an undefined dose of continuous regimen CEE for 36 months with a fully compliant proportion of patients under 50% (Table 1 and Table S2) [23,25]. No difference was found between HT users and the placebo [25]. In a sub-analysis, Maxwell and coworkers in the HT-treated group found that the Black population had a significantly increased risk of cancer recurrence compared to the White population (five recurrences in 56 subjects vs. eight in 521 subjects) (Table S2) [23]. The genetic differences implicated in estrogen biosynthesis, metabolism, and related pathways may contribute to this increased risk of tumor recurrence in the sub-population of Black women treated with HT [23,32]. In fact, Black Americans are generally diagnosed with more aggressive endometrial or breast cancer subtypes, like the estrogen-receptor-negative breast cancer, than the European population [32,33]. Moreover, Black Americans and indigenous African women are more likely to be diagnosed with larger uterine fibroids at an earlier age than the European population [34]. In addition, African women have higher serum concentrations of estrogen, as well as different profiles of hormone-related factors than the European population [32]. It is reasonably hypothesized that these racial disparities are partly due to different estrogen metabolisms and related hormonal factors [32,34]. The second study that used estrogen alone was the retrospective cohort study by Creasman and coworkers that treated the women for a median duration of 26 months with 0.625 or 1.25 mg/d of CEE using a cyclic regimen [29]. Creasman and coworkers found a protective effect of estrogen-only HT, but no correction of immortal time bias was performed, leading to a possible overestimation of the HT treatment benefit. Furthermore, these results should be interpreted with caution since combined HT and cyclic regimens were used only in observational studies, and these studies are affected by immortal time bias and the previously discussed biases. Moreover, only a portion of HT users in three of these studies was treated with a combined HT [20,26,28]. Hence, the observed differences between estrogen-only and combined HT can be related to the different study types, rather than the HT type (estrogen-only treatment vs. combined HT or continuous vs. cyclic regimen). Although uterine-preserving therapy with progesterone may be used in young women with endometrial cancer who desire fertility preservation [35,36], the use of combined HT in EC patients merits further research to better clarify the possible advantages.

The majority of studies showing that HT did not increase the risk of EC recurrences had a population restricted to those with stage I and II tumors [21,22,24,25,27,28,29]. Whilst Suriano and coworkers also included stage III [26], the number of patients was limited (only four). Therefore, there is no evidence that HT is safe after treatment for advanced EC. In fact, any residual cancer cells could be potentially stimulated by an estrogen-based HT.

Most of the considered studies used a standard dose of 0.625 mg/d CEE [24,26], with many exceptions where the dosage, in an undefined percentage of patients, was up to 1.25 mg/d CEE [27,28,29]. Considering these two groups of studies, no differences in reducing the risk of EC recurrence were found. In addition, in other studies, the drugs used were different, or the dosage was not specified.

Given that in some studies, oral/transdermal and vaginal estrogen [26,27] were considered to represent systemic use, because topical estrogen can increase systemic estradiol levels [37], all of these administration routes were assessed. However, the vaginal topical treatment was limited to a proportion of patients within these studies—specifically, in the study by Creasman and coworkers, 72% of cases [29]; in Chapman and Coworkers, 1.6% [27]; and in Suriano and coworkers, the proportion of cases was not specified [26]. Despite the potential involvement of many confounding factors, we found that in studies where the vaginal route was also used, both OR and HR were slightly more protective than in studies where only an oral administration was considered (Figure 3C and Figure 4B).

The pooled HT mean duration was 43.40 months (95% CI 36.52 to 50.27). No differences were found between the studies subgrouped by HT median duration (Figure S2C,D), probably because the mean duration of HT exceeded the interval of the majority of EC recurrences.

Since the disease-free interval varied significantly among the included studies, it could be considered a source of bias, particularly if the disease-free interval is longer than the median time of a recurrence. In three of the included studies, HT was initiated within 5 months after surgery [21,23,24,25]. However, the majority of the studies had a pooled disease-free interval of 21 months, and Chapman and coworkers had a mean of 87 months [26,27,28,29], so there was a high proportion of women at low risk of experiencing an EC recurrence.

The only one RCT published to date and presented in two different articles [23,25] solves many of the inherent limitations of observational studies and shows the most unbiased data in the current literature. However, this RCT is subject to some important limitations. Firstly, the target sample size was not achieved due to poor patient recruitment, attributed to a lack of HT acceptance after a Women’s Health Initiative publication [38]. Secondly, the enrolled population was at a low risk of cancer recurrence anyway, thus requiring the study to be unfeasibly large to detect differences in disease-free survival (i.e., under-powered study).

Estrogen promotes viability and decreases autophagy in estrogen receptor α positive Ishikawa cells, but not in KLE cells with defective estrogen receptors [39]. It is known that estrogen increases mitosis rates that can trigger mutations due to replication failures [40]. Therefore, investigations into the ways that endometrial cancer could be treated by inhibiting estrogen suggested pathways by treatment with progesterone. However, although uterine-sparing therapy with progesterone in young women was found to be promising [35,36], the use of hormone adjuvant therapy was not effective in endometrial cancer patients after surgical treatment [41]. This lack of response to hormone therapy differs from breast cancer, where the literature demonstrates the benefit of adjuvant hormonal therapy in reducing recurrences and improving survival [42]. This is a fascinating topic, which could be linked to the loss of expression of estrogen receptors in metastatic endometrial tissue from estrogen positive primary tumors [43] and indicating the activation of alternative oncogenic pathways. Furthermore, there is no demonstration of an in vivo estrogen effect on any residual microscopic cancer cells [44]. Hence, it is hypothesized that estrogen can act as a tumor promoter in the presence of orthotopic endometrial tissue without having harmful effects after neoplastic transformation of endometrial tissue [26] or, more likely, cancer progression post-surgical uterus removal probably depends on the presence of residual circulating tumor cells and not on estrogen effects [6,26]. Notwithstanding the previous hypothesis, the mechanisms underlying the relationship between HT and EC recurrence are not yet completely understood, and therefore further research is needed.

### 4.3. Comparison with the Literature

In line with these findings, similar results were obtained recently in a meta-analysis by Shim and coworkers [6]; however, they considered two studies with overlapping data in their pooled analysis [26,27]. In addition, two new cohort studies were published after 2014, and in the previous article, a time-to-event analysis was not performed [6]. Moreover, Shim and coworkers did not include the article published by Maxwell and coworkers, and thus failed to factor in ethnicity, which appeared in our results to be an important confounding factor [6,23].

### 4.4. Strengths and Weaknesses

The major strength of this study is the analysis of EC recurrences by means of a time-to-event pooled analysis. In addition, recent large cohort studies are included. However, the results of this meta-analysis should be interpreted with caution because of a number of limitations. First of all, language bias may have occurred because articles published in languages other than English, Italian, German, French, or Spanish were not included in the review; as HT in EC survivors appears to be an issue facing many people throughout the world, some relevant studies published in other languages may have been excluded. Secondly, the majority of studies were observational, which could have impacted on the accuracy of collecting data and which also presented issues related to confounding factors that cannot be addressed in a meta-analysis (such as immortal time bias). Although some of the studies we reviewed adjusted for potential confounding factors, the possibility of residual effects cannot be ruled out. Thirdly, by selecting only the available full text articles, the findings might have been influenced by the ‘full text bias’ [45]. Fourthly, most of the literature about this argument was published in the last few decades of the previous century or the early years of this century, and consequently does not consider the advances made in managing endometrial cancer in recent years [46]. Finally, publication bias, by its nature, is difficult to detect and could not be completely ruled out, even if shown to be negative, because small studies showing a detrimental effect of treatment are unlikely to be published (the alleged “small-study effect”) [47]. This would potentially underestimate the risk of recurrence in EC survivors using HT. The Egger test is better at detecting the presence of the small-study effect than the rank correlation test, but it has inadequate power if less than 10 studies are tested [47]. In our case, both tests failed to detect significant bias. Subsequently, the results of this meta-analysis should be interpreted within the context of its limitations.

### 4.5. Generalisability

Consistent methodological weaknesses limit the generalizability of these findings and currently preclude firm conclusions. No conclusion could be reached regarding advanced EC stages, because although one article included stage III EC cases, there was still not a large enough sample size to allow us to reach any conclusion [26], and even more so a recommendation. Additionally, the wide heterogeneity of HT therapies prevents us from reaching any definite conclusions.

### 4.6. Relevance of the Findings

The relevance of these findings to women treated for EC is to allow patients displaying menopausal symptoms greater confidence when opting for HT treatment, by conducting a systematic evaluation of the current data available to us. In fact, many studies in the literature are underpowered, including the only RCT (which was in fact canceled for this reason), and thanks to this meta-analysis, we can get a clearer idea of what the effects of HT are (our observation being that there are greater benefits than unfavorable effects in EC survivors). These data thus allow clinicians to make a more evidence-based decision regarding treatment options, rather than one based on the presumption and experience of the single clinician.

### 4.7. Unanswered Questions and Future Research

Potentially confounding factors that emerged were ethnicity, type of HT, the age of the patient, disease-free interval, immortal time bias, and cancer characteristics (i.e., tumor staging, grading, histological, and molecular subtypes of EC). These factors were not able to be fully explored with existing data and should be considered as potentially confounding aspects in future RCT or in large multicentric prospective observational studies. In addition, as cancer recurrences are time-dependent events, they should be managed with proper analysis, especially in observational studies, to reduce possible biases related to the time of event occurrence (e.g., Kaplan–Meier, Cox regression, or other appropriate methods). Furthermore, considering all these factors, there is the necessity to clearly identify which EC survivors could benefit from HT, in order to offer safe and effective management of post-menopausal symptoms. Fortunately, the early stages of endometrial cancer have a very favorable prognosis with a recurrence-free survival of 90% at 5 years [48]. However, this does also mean there will be a low number of negative events, which implies the need for large sample sizes that can be reached only with large multicentric randomized controlled trials or with widely adopted international registries, such as RIETE (Registro Informatizado de la Enfermedad ThromboEmbolica) [49].

## 5. Conclusions

Summing up, these results suggest that, in general, HT use does not increase the incidence of EC recurrences in women treated for early stages EC (FIGO I or II), except for Black American women where a significantly increased recurrence risk is evident. Therefore, the positive effects of HT outweigh eventual risks, with the exception of Black American women. Future studies with a longer follow-up are necessary to better clarify the long-term effects of HT in this set of individuals. From the data currently available in the literature, no definite conclusions can be reached on HT type, mode, or duration.

## Figures and Tables

**Table 1:** Description of the included studies.

Study	Location	Period	Study Type	HRT Type	HRT Notes
Barakat 2006/Maxwell 2008	USA	1997–2003	RCT	Continuous HRT (E)	CEEs (dose not specified)
Cho 2019	Korea	2010–2013	NRS (CS)	Mix (included continous EP)	Estrogen only, Estrogen plus progesterone, Tibolone, Progesterone only
Ayhan 2006	Turkey	1992–2001	NRS (CC)	Continuous HRT (EP)	CEEs (0.625 mg/d) plus MPA (2.5 mg/d)
Lim 2018	Korea	2006–2014	NRS (CS)	Continuous HRT (EP)	E2/DRSP (1 mg/2 mg)
Chapman 1996	USA	1982–1994	NRS (CC)	Mix (included continous EP)	CEEs (0.625 or 1.25 mg/d) with or without progesterone (2.5 mg/d)
Creasman 1986	USA	1975–1980	NRS (CS)	Cyclic HRT (E)	CEEs only (0.625 or 1.25 mg/d)
Lee 1990	USA	1975–1985	NRS (CC)	Mix (included Cyclic EP)	CEEs (0.625 or 1.25 mg/d) with or without progestin
Suriano 2001	USA	1984–1998	NRS (CS)	Mix (included continous EP)	CEEs (0.625 mg/d) with or without MPA (2.5 mg/d)
Arteaga-Gómez 2011	Mexico	2000–2008	NRS (CS)	Mix (included continous EP)	Estrogen only, Tibolone

Acronyms. RCT = randomized controlled trial; NRS = non-randomized study; CC = case control; CS = cohort study; CEE = conjugated equine estrogen; MPA = medroxyprogesterone acetate; E2 = estradiol; DRSP = drospirenone.

## Data Availability

The data that support the findings of this study are available, but restrictions apply to the availability of these data, which was used under license for the current study, and so are not publicly available. Data are however available from the authors upon reasonable request and with permission of the Internal Review Board.

## Supplementary Materials

The following are available online at https://www.mdpi.com/article/10.3390/jcm10143165/s1, Figure S1: Panel (A) Mean difference in patient age between HT and controls. Panel (B) Mean follow up time. Panel (C) Mean difference in follow up time between patients treated with HT and controls, Figure S2: Panel (A) Forest plot of hazard ratio (HR) meta-analysis for disease free survival/recurrence risk showing pooled random effect model subgroup analysis by disease free interval time in months before starting HT. Panel (B) Forest plot of odds ratio (OR) meta-analysis for recurrence risk showing pooled fixed-effect model subgroup analysis by disease free interval time in months before starting HT. Panel (C) Forest plot of hazard ratio (HR) meta-analysis for disease free survival/recurrence risk showing pooled random effect model subgroup analysis by duration of HT. Panel (D) Forest plot of odds ratio (OR) meta-analysis for recurrence risk showing pooled fixed-effect model subgroup analysis by duration of HT, Figure S3: Forest plot of hazard ratio (HR) meta-analysis for disease free survival/recurrence risk showing pooled random effect model subgroup analysis by tumor stage, Table S1: Summary of database queries. Table S2: Included studies summary showing inclusion/exclusion criteria, matched variables (for case-control studies), and adjusted variables in multivariate analysis. Besides, this table shows information about HT compliance, timing, duration, and other study characteristics. List S1: Included and excluded studies.

## Abbreviations

BMI	body mass index
CA	cancer antigen
CC	case control
CEBM	Centre for Evidence-Based Medicine
CEE	conjugated equine estrogen
CI	confidence interval
CS	cohort study
DRSP	drospirenone
E2	estradiol
EC	endometrial cancer
EMBASE	excerpta medica database
FIGO	International Federation of Gynecology and Obstetrics (Fédération Internationale de Gynécologie et d’Obstétrique)
HR	hazard ratio
HT	hormone therapy
ICTRP	International Clinical Trials Registry Platform
IQR	interquartile range
LN	lymph node
MPA	medroxyprogesterone acetate
NRS	non-randomized study
OR	odds ratio
PRISMA	Preferred Reporting Items for Systematic Reviews and Meta-Analyses
RCT	randomised controlled trial
RR	relative risk
RT	radiotherapy
