# Supplementary material for: Hormone Replacement Therapy in Endometrial Cancer Survivors: A Meta-Analysis
Source: J Clin Med. 2021 Jul 18;10(14):3165. doi: 10.3390/jcm10143165 (PMC8303659; doi:10.3390/jcm10143165)
Supplement: Supplementary file 1 [file jcm-10-03165-s001.zip › jcm-1267164-supplementary.pdf]

Table S1- Summary of database queries.

Database	Query	Date	Number of items
PubMed/Medline	("hormone replacement therapy" OR "hormone therapy" OR "estrogen replacement therapy" OR (menopausal hormone replacement therapy) OR (menopausal estrogen and estrogen-progestin replacement therapy) OR "HRT" OR "HT" OR "ERT" OR "MHRT" OR "MRT") AND ((endometrial OR endometrium) AND (neoplasm* OR carcinoma* OR cancer))	06.07.2021	2047
Scopus	TITLE-ABS-KEY (("hormone replacement therapy" OR "hormone therapy" OR "estrogen replacement therapy" OR (menopausal hormone replacement therapy) OR (menopausal estrogen and estrogen-progestin replacement therapy) OR "HRT" OR "HT" OR "ERT" OR "MHRT" OR "MRT") AND ((endometrial OR endometrium) AND (neoplasm* OR carcinoma* OR cancer)))	06.07.2021	4573
Cochrane Central Register of Controlled Trials	("hormone replacement therapy" OR "hormone therapy" OR "estrogen replacement therapy" OR (menopausal hormone replacement therapy) OR (menopausal estrogen and estrogen-progestin replacement therapy) OR "HRT" OR "HT" OR "ERT" OR "MHRT" OR "MRT") AND ((endometrial OR endometrium) AND (neoplasm* OR carcinoma* OR cancer))	06.07.2021	301
Cochrane Central Register of Reviews	("hormone replacement therapy" OR "hormone therapy" OR "estrogen replacement therapy" OR (menopausal hormone replacement therapy) OR (menopausal estrogen and estrogen-progestin replacement therapy) OR "HRT" OR "HT" OR "ERT" OR "MHRT" OR "MRT") AND ((endometrial OR endometrium) AND (neoplasm* OR carcinoma* OR cancer))	06.07.2021	157
EMBASE	("hormone replacement therapy" OR "hormone therapy" OR "estrogen replacement therapy" OR (menopausal hormone replacement therapy) OR (menopausal estrogen and estrogen-progestin replacement therapy) OR "HRT" OR "HT" OR "ERT" OR "MHRT" OR "MRT") AND ((endometrial OR endometrium) AND (neoplasm* OR carcinoma* OR cancer))	06.07.2021	4433
Web of science	("hormone replacement therapy" OR "hormone therapy" OR "estrogen replacement therapy" OR (menopausal hormone replacement therapy) OR (menopausal estrogen and estrogen-progestin replacement therapy) OR "HRT" OR "HT" OR "ERT" OR "MHRT" OR "MRT") AND ((endometrial OR endometrium) AND (neoplasm* OR carcinoma* OR cancer))	06.07.2021	2204
Clinicaltrials.gov	("hormone therapy" OR "hormone replacement therapy" OR menopause OR estrogen) AND ("endometrial neoplasms" OR "endometrial cancer") AND Limited to studies with Female Participants	06.07.2021	103
International Clinical Trials Registry Platform (ICTRP)	("hormone therapy" OR "hormone replacement therapy" OR menopause OR estrogen) AND ("endometrial neoplasms" OR "endometrial cancer") AND Limited to studies with Female Participants	06.07.2021	8

* Is a wildcard it means neoplasm or neoplasms ect.

Supplemental Table 2- Included studies summary showing inclusion/exclusion criteria, matched variables (for case-control studies), and adjusted variables in multivariate analysis. Besides, this table shows information about HT compliance, timing, duration, and other study characteristics.

**Labels**	**Type**	**Inclusion criteria**	**Matching**	**Exclusion criteria**	**Multivariate analysis**	**Compliance to HT or HT use verification**	**Timing of HT initiation**	**Number controls/ HT**	**Number recurrences in controls / HT**	**Treatment duration/ follow-up (months) (†)**	**Study conclusions**
**RCT**											
Barakat 2006/Maxwell 2008	Prospective	Stage I or II endometrial cancer (adenocarcinoma) with surgical staging, type of surgery, indication for HT, HT timing, negative imaging and laboratory assessment	--- (§)	Other invasive malignancies, liver disease, thromboembolic disease, other sex hormonal therapy	Age, BMI, and tumor grade	Monitored using unused tablet counts, patient logs, patient inquiries, and adverse effects<br>HT: 41.1% fully compliant<br>Placebo: 50.1% fully compliant	Same as placebo (<5 months)	All<br>618/618<br>White (¶)<br>528/521<br>Blacks (¶)<br>54/56	All<br>12/14<br>White (¶)<br>12/8<br>Blacks (¶)<br>0/5	36/36	1) Not conclusive, but low recurrence and low new malignancy;<br>2) Not in favor to HT in Black patients
**Case-control studies**											
Ayhan 2006	Retrospective (*)	Cases: stage I or II endometrial cancer with surgical staging, and HT timing<br>Controls: matched with cases considering tumor characteristics and treatment period	Age; parity; systemic diseases; preoperative use of HT; stage, grade, depth of myometrial invasion; LVSI; tumoral size; resected LN number; use of adjuvant RT; and mean follow-up period	RT before surgery, initial surgery at another center, stage III or IV disease, histology other than endometrioid type, second malignancy, and general contraindications of HT	NA	NA	<1-2 months	52/50	1/0	49.1/49.1 HT and 53.2 controls	In favor of HT in patients after surgery
Chapman 1996	Retrospective	Cases: stage I or II endometrial cancer<br>Controls: matched with cases considering physician and treatment period	Same time interval, same physicians	Advanced-stage disease, histologic diagnosis other than adenocarcinoma, simultaneous ovarian carcinoma, other malignancies	NA	Chart review and personal communication	Mean 87.25 months	61/62	6/2	39.5/HT 57.1 and 39.1 controls	Not conclusive, but data suggest better disease-free survival in the estrogen replacement therapy group
Lee 1990	Retrospective	Cases: stage I endometrial cancer (adenocarcinoma)<br>Controls: matched considering treatment period	Same time interval	Stage >I, histology	NA	Chart review, tumor registry, and personal communication	Mean 22.66 months	99/44	8/0	64/64(‡)	In favor of HT in patients after surgery

Labels	Type	Inclusion criteria	Matching	Exclusion criteria	Multivariate analysis	Compliance to HT or HT use verification	Timing of HT initiation	Number controls/ HT	Number recurrences in controls / HT	Treatment duration/ follow-up (months) (†)	Study conclusions
**Cohort studies**											
Cho 2019	Retrospective	Newly diagnosed endometrial cancer with surgical staging	--- (§)	No surgical staging and type of surgery	Age, assessment of lymph nodes, and adjuvant therapy	National insurance database	NA	4820/847	396/50	23.06/47.4	In favor of HT in patients after surgery
Lim 2018	Retrospective	Endometrial cancer (endometrioid) with surgical staging and HT timing	--- (§)	Incomplete surgical staging, stage >II, non-endometrioid carcinoma, synchronous ovarian cancer, and incomplete medical records	Stage, tumor diameter, receptor status, adjuvant therapy, and preoperative CA 125	NA	<2 months	116/58	3/1	66.76/64(‡)	In favor of HT in patients after surgery
Creasman 1986	Retrospective	Stage I or II endometrial cancer with primary treatment	--- (§)	NA	Age, substage, grade, depth of invasion, pelvic node status, peritoneal cytology, estrogen receptor status, and progesterone receptor status	Personal communication	Mean 28.02 months<br><br>Median 15 months (min 8 – max 81 months)	174/47	26/1	26(‡)/32(‡)	In favor of HT in patients after surgery
Suriano 2001	Retrospective	Endometrial cancer with surgical staging and indication for HT	Age and stage (for case control)	Surgical stage IV disease, sarcomatous histology, follow-up <12 months, inability to confirm HT use, concurrent tamoxifen use, and absence of appropriate surgical staging	Age and preoperative use of hormone therapy	NA	Mean 12.93 months (subgroup analysis <6 months)	Cohort: 119/130 Case-control: 75/75	Cohort: --- Case-control: 11/2	NA/83 HT and 43.3 controls	In favor of HT in patients after surgery
Arteaga-Gómez 2011	Retrospective	Stage I or II endometrial cancer with surgical staging, and indication for HT	--- (§)	Stage >II, no indication for HT	NA	Chart review (menopausal outpatients facility)	NA	18/11	1/0	40.05/57.1	HT can be safely administered in stages I and II without affecting the disease-free period

Acronyms. LN= lymph node; RT= radiotherapy; BMI= body mass index; CA= cancer antigen; LVSI = lymphovascular space invasion; NA= not available; HT: hormone therapy.
Legend: (*) claimed as to be prospective, reclassified as retrospective from a prospective database; (†) presented as mean value except where otherwise specified; (‡) median; (§) not applicable; (¶) Maxwell et al. excluded the non-white and non-black women.

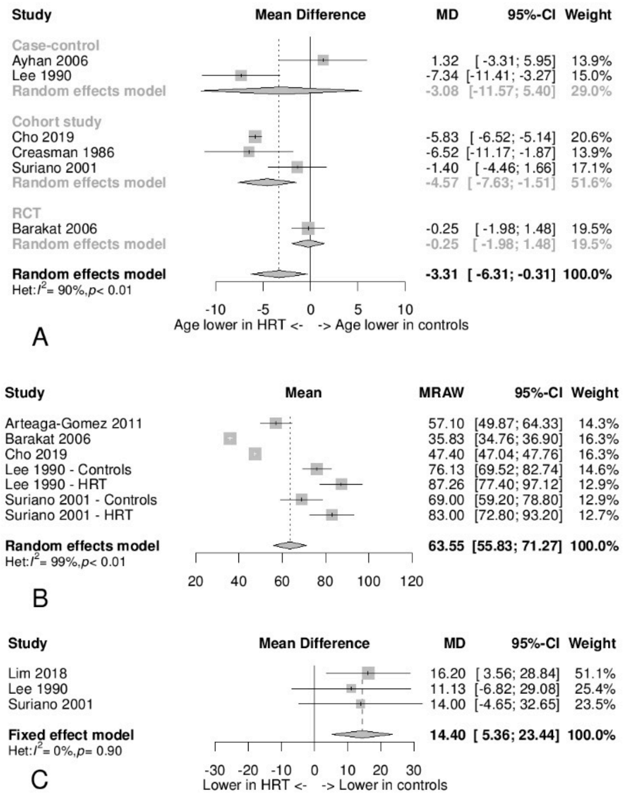
Study
Mean Difference
MD
95%-CI
Weight
Case-control
Ayhan 2006
1.32
[-3.31; 5.95]
13.9%
Lee 1990
-7.34
[-11.41; -3.27]
15.0%
Random effects model
-3.08
[-11.57; 5.40]
29.0%
Cohort study
Cho 2019
-5.83
[-6.52; -5.14]
20.6%
Creasman 1986
-6.52
[-11.17; -1.87]
13.9%
Suriano 2001
-1.40
[-4.46; 1.66]
17.1%
Random effects model
-4.57
[-7.63; -1.51]
51.6%
RCT
Barakat 2006
-0.25
[-1.98; 1.48]
19.5%
Random effects model
-0.25
[-1.98; 1.48]
19.5%
Random effects model
-3.31
[-6.31; -0.31]
100.0%
Het: I2= 90%, p< 0.01
-10
-5
0
5
10
Age lower in HRT <-
-> Age lower in controls
A
Study
Mean
MRAW
95%-CI
Weight
Arteaga-Gomez 2011
57.10
[49.87; 64.33]
14.3%
Barakat 2006
35.83
[34.76; 36.90]
16.3%
Cho 2019
47.40
[47.04; 47.76]
16.3%
Lee 1990 - Controls
76.13
[69.52; 82.74]
14.6%
Lee 1990 - HRT
87.26
[77.40; 97.12]
12.9%
Suriano 2001 - Controls
69.00
[59.20; 78.80]
12.9%
Suriano 2001 - HRT
83.00
[72.80; 93.20]
12.7%
Random effects model
63.55
[55.83; 71.27]
100.0%
Het: I2= 99%, p< 0.01
20
40
60
80
100
120
B
Study
Mean Difference
MD
95%-CI
Weight
Lim 2018
16.20
[3.56; 28.84]
51.1%
Lee 1990
11.13
[-6.82; 29.08]
25.4%
Suriano 2001
14.00
[-4.65; 32.65]
23.5%
Fixed effect model
14.40
[5.36; 23.44]
100.0%
Het: I2= 0%, p= 0.90
-30
-20
-10
0
10
20
30
Lower in HRT <-
-> Lower in controls
C

Figure S1

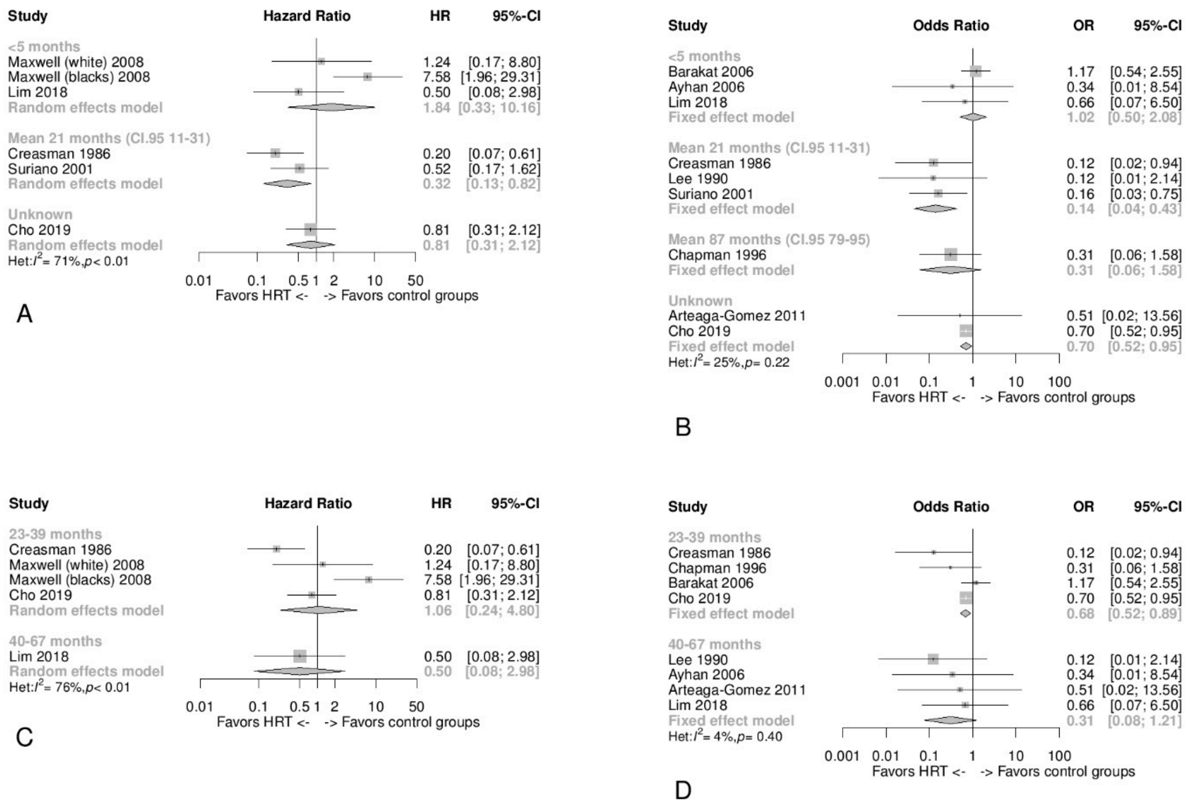
Study
Hazard Ratio
HR
95%-CI
<5 months
Maxwell (white) 2008 1.24 [0.17; 8.80]
Maxwell (blacks) 2008 7.58 [1.96; 29.31]
Lim 2018 0.50 [0.08; 2.98]
Random effects model 1.84 [0.33; 10.16]
Mean 21 months (CI.95 11-31)
Creasman 1986 0.20 [0.07; 0.61]
Suriano 2001 0.52 [0.17; 1.62]
Random effects model 0.32 [0.13; 0.82]
Unknown
Cho 2019 0.81 [0.31; 2.12]
Random effects model 0.81 [0.31; 2.12]
Het: I^2= 71%, p< 0.01
0.01 0.1 0.5 1 2 10 50
Favors HRT <- -> Favors control groups
A
Study
Odds Ratio
OR
95%-CI
<5 months
Barakat 2006 1.17 [0.54; 2.55]
Ayhan 2006 0.34 [0.01; 8.54]
Lim 2018 0.66 [0.07; 6.50]
Fixed effect model 1.02 [0.50; 2.08]
Mean 21 months (CI.95 11-31)
Creasman 1986 0.12 [0.02; 0.94]
Lee 1990 0.12 [0.01; 2.14]
Suriano 2001 0.16 [0.03; 0.75]
Fixed effect model 0.14 [0.04; 0.43]
Mean 87 months (CI.95 79-95)
Chapman 1996 0.31 [0.06; 1.58]
Fixed effect model 0.31 [0.06; 1.58]
Unknown
Arteaga-Gomez 2011 0.51 [0.02; 13.56]
Cho 2019 0.70 [0.52; 0.95]
Fixed effect model 0.70 [0.52; 0.95]
Het: I^2= 25%, p= 0.22
0.001 0.01 0.1 1 10 100
Favors HRT <- -> Favors control groups
B
Study
Hazard Ratio
HR
95%-CI
23-39 months
Creasman 1986 0.20 [0.07; 0.61]
Maxwell (white) 2008 1.24 [0.17; 8.80]
Maxwell (blacks) 2008 7.58 [1.96; 29.31]
Cho 2019 0.81 [0.31; 2.12]
Random effects model 1.06 [0.24; 4.80]
40-67 months
Lim 2018 0.50 [0.08; 2.98]
Random effects model 0.50 [0.08; 2.98]
Het: I^2= 76%, p< 0.01
0.01 0.1 0.5 1 2 10 50
Favors HRT <- -> Favors control groups
C
Study
Odds Ratio
OR
95%-CI
23-39 months
Creasman 1986 0.12 [0.02; 0.94]
Chapman 1996 0.31 [0.06; 1.58]
Barakat 2006 1.17 [0.54; 2.55]
Cho 2019 0.70 [0.52; 0.95]
Fixed effect model 0.68 [0.52; 0.89]
40-67 months
Lee 1990 0.12 [0.01; 2.14]
Ayhan 2006 0.34 [0.01; 8.54]
Arteaga-Gomez 2011 0.51 [0.02; 13.56]
Lim 2018 0.66 [0.07; 6.50]
Fixed effect model 0.31 [0.08; 1.21]
Het: I^2= 4%, p= 0.40
0.001 0.01 0.1 1 10 100
Favors HRT <- -> Favors control groups
D

Figure S2

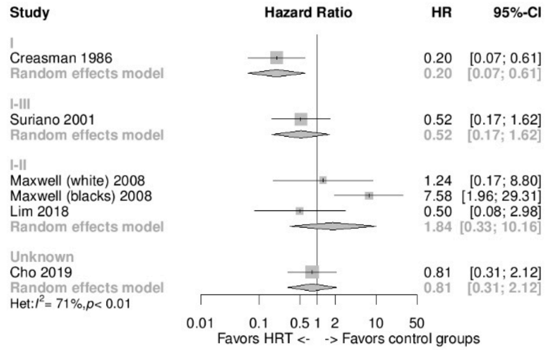
Study
Hazard Ratio
HR
95%-CI
I
Creasman 1986
0.20
[0.07; 0.61]
Random effects model
0.20
[0.07; 0.61]
I-III
Suriano 2001
0.52
[0.17; 1.62]
Random effects model
0.52
[0.17; 1.62]
I-II
Maxwell (white) 2008
1.24
[0.17; 8.80]
Maxwell (blacks) 2008
7.58
[1.96; 29.31]
Lim 2018
0.50
[0.08; 2.98]
Random effects model
1.84
[0.33; 10.16]
Unknown
Cho 2019
0.81
[0.31; 2.12]
Random effects model
0.81
[0.31; 2.12]
Het: I^2= 71%, p< 0.01
0.01
0.1
0.5
1
2
10
50
Favors HRT <-
-> Favors control groups

Figure S3

List S1

INCLUDED STUDIES

Studies that fulfilled the requirements.

References

[1] Barakat RR, Bundy BN, Spirtos NM, et al. Randomized Double-Blind Trial of Estrogen Replacement Therapy versus Placebo in Stage I or II Endometrial Cancer: A Gynecologic Oncology Group Study. J Clin Oncol. 2006;24:587–592.
[2] Maxwell GL, Tian C, Risinger JI, et al. Racial Disparities in Recurrence among Patients with Early-Stage Endometrial Cancer: Is Recurrence Increased in Black Patients Who Receive Estrogen Replacement Therapy? Cancer. 2008;113:1431–1437.
[3] Cho HW, Ouh YT, Lee JK, et al. Effects of Hormone Therapy on Recurrence in Endometrial Cancer Survivors: A Nationwide Study Using the Korean Health Insurance Review and Assessment Service Database. J Gynecol Oncol. 2019;30:e51.
[4] Ayhan A, Taskiran C, Simsek S, et al. Does Immediate Hormone Replacement Therapy Affect the Oncologic Outcome in Endometrial Cancer Survivors? Int J Gynecol Cancer. 2006 Mar-Apr;16:805–808.
[5] Lim S, Kim YH, Lee KB, et al. The Influence of Hormone Therapy with Drospirenone-Estradiol on Endometrioid Type Endometrial Cancer Patients. J Gynecol Oncol. 2018;29:e72.
[6] Chapman JA, DiSaia PJ, Osann K, et al. Estrogen Replacement in Surgical Stage I and II Endometrial Cancer Survivors. Am J Obstet Gynecol. 1996;175:1195–1200.
[7] Creasman WT, Henderson D, Hinshaw W, et al. Estrogen Replacement Therapy in the Patient Treated for Endometrial Cancer. Obstet Gynecol. 1986;67:326–330.
[8] Lee RB, Burke TW, Park RC. Estrogen Replacement Therapy Following Treatment for Stage I Endometrial Carcinoma. Gynecol Oncol. 1990;36:189–191.
[9] Suriano KA, McHale M, McLaren CE, et al. Estrogen Replacement Therapy in Endometrial Cancer Patients: A Matched Control Study. Obstet Gynecol. 2001;97:555–560.
[10] Arteaga-Gómez AC, Castellanos-Barroso G, Colin-Valenzuela A, et al. Hormone Therapy Effect in Postmenopausal Women with History of Endometrial Cancer. Ginecol Obstet Mex. 2011;79:11.

EXCLUDED STUDIES

Studies without a control group or without the selected outcomes or exposures [1–13].

Editorials / comments / letters [14–32].

Reviews or other publications without original data [33–89].

References

[1] Lindh-Åstrand L, Hoffmann M, Fredrikson M, et al. Use of Hormone Therapy (HT) among Swedish Women with Contraindications - A Pharmacoepidemiological Cohort Study. Maturitas. 2019;123:55–60.
[2] Gitsch G, Hanzal E, Jensen D, et al. Endometrial Cancer in Premenopausal Women 45 Years and Younger. Obstet Gynecol. 1995;85:504–508.
[3] Vistad I, Bjørge L, Solheim O, et al. A National, Prospective Observational Study of First Recurrence after Primary Treatment for Gynecological Cancer in Norway. Acta Obstet Gynecol Scand. 2017;96:1162–1169.
[4] Greenwald ZR, Huang LN, Wissing MD, et al. Does Hormonal Therapy for Fertility Preservation Affect the Survival of Young Women with Early-Stage Endometrial Cancer? Cancer. 2017;123:1545–1554.
[5] Woolas RP, Hammond IG, McCartney AJ. Endometrial Cancer before Age 35 and Subsequent Use of Hormone Replacement Therapy. Journal of Obstetrics and Gynaecology. 1993;13:468–470.
[6] Rozenberg S, Vasquez JB. Estrogen Replacement Therapy in Patients with Endometrial Cancer: Prescription Attitude of Belgian Gynecologists. Maturitas. 2000;35:125–128.
[7] Hein A, Thiel FC, Bayer CM, et al. Hormone Replacement Therapy and Prognosis in Ovarian Cancer Patients. Eur J Cancer Prev. 2013;22:52–58.
[8] Comert GK, Turkmen O, Kar I, et al. Hormone Therapy Following Surgery in Low-Grade Endometrial Stromal Sarcoma: Is It Related to a Decrease in Recurrence Rate? J Chin Med Assoc. 2019;82:385–389.
[9] Jacob L, Kostev K, Kalder M. Prescription of Hormone Replacement Therapy Prior to and after the Diagnosis of Gynecological Cancers in German Patients. J Cancer Res Clin Oncol. 2020;146:1567–1573.
[10] Li C, Samsioe G, Iosif C. Quality of Life in Endometrial Cancer Survivors. Maturitas. 1999;31:227–236.
[11] Hein A, Schneider MO, Renner SK, et al. Risk of Postmenopausal Hormone Therapy and Patient History Factors for the Survival Rate in Women with Endometrial Carcinoma. Arch Gynecol Obstet. 2020;301:289–294.
[12] Vinklerová P, Minář L, Felsinger M, et al. The Role of Hormonal Therapy in Patients with Uterine Carcinoma. Ceska Gynekol;83:263–270.
[13] Chambers LM, Herrmann A, Michener CM, et al. Vaginal Estrogen Use for Genitourinary Symptoms in Women with a History of Uterine, Cervical, or Ovarian Carcinoma. Int J Gynecol Cancer. 2020;30:515–524.
[14] Ulrich L. HRT after Endometrial Cancer–Is It Safe? Maturitas. 2014;79:237–238.
[15] Jaffe RB. Endometrial Cancer and Hormone-Replacement Therapy in the Million Women Study:. Obstet Gynecol Surv. 2005;60:595–597.
[16] Wiegratz I, Kuhl H. Endometrial Cancer and Hormone-Replacement Therapy. Lancet. 2005;366:201–202.
[17] McKnight B, Voigt LF, Beresford SA, et al. Re: Estrogen-Progestin Replacement Therapy and Endometrial Cancer. J Natl Cancer Inst. 1998;90:164–166.
[18] Rose P. Altering HRT for Individual Needs, Endometrial Cancer & Estrogen Therapy. ACOG Clin Rev. 1998; 3:13.
[19] Bryant GW. Administration of Estrogens to Patients with a Previous Diagnosis of Endometrial Adenocarcinoma. South Med J. 1990;83:725–726.
[20] Genazzani AR, Gadducci A, Gambacciani M, et al. Controversial Issues in Climacteric Medicine II. Hormone Replacement Therapy and Cancer. Maturitas. 2001;40:117–130.
[21] ACOG. Estrogen Replacement Therapy and Endometrial Cancer: ACOG Committee Opinion: Committee on Gynecologic Practice Number 126 - August 1993 (Replaces No. 80, February 1990). International Journal of Gynecology & Obstetrics. 1993;43:89–89.
[22] ACOG. Estrogen Replacement Therapy and Endometrial Cancer: ACOG Committee Opinion: Committee on Gynecologic Practice Number 80 - February 1990. International Journal of Gynecology & Obstetrics. 1991; 36:76–76.
[23] Lee RB, Burke TW, Park RC. Estrogen Replacement Therapy Following Treatment for Stage I Endometrial Carcinoma:. Obstetrical & Gynecological Survey. 1990;45:558–559.
[24] Rees M, Angioli R, Coleman RL, et al. European Menopause and Andropause Society (EMAS) and International Gynecologic Cancer Society (IGCS) Position Statement on Managing the Menopause after Gynecological Cancer: Focus on Menopausal Symptoms and Osteoporosis. Maturitas. 2020;134:56–61.
[25] Rees M, Angioli R, Coleman RL, et al. European Menopause and Andropause Society (EMAS) and International Gynecologic Cancer Society (IGCS) Position Statement on Managing the Menopause after Gynecological Cancer: Focus on Menopausal Symptoms and Osteoporosis. Int J Gynecol Cancer. 2020; 30:428–433.

[26] Weinstein L. Hormonal Therapy in the Patient with Surgical Menopause. Obstet Gynecol. 1990; 75:47S–50S; discussion 51S–52S.
[27] Ortmann O, Emons G, Tempfer C. Hormonersatztherapie nach hormonabhängigen Krebserkrankungen gemäß S3-Leitlinie. Gynäkologe. 2020;53:156–160.
[28] Ortmann O, Emons G, Tempfer C. Hormonersatztherapie nach hormonabhängigen Krebserkrankungen gemäß S3-Leitlinie. Gynäkologische Endokrinologie. 2020;18:8–13.
[29] International Menopause Society. Hormone Replacement Therapy and Cancer. Gynecological Endocrinology. 2001;15:453–465.
[30] Panay N, Fenton A. Iatrogenic Menopause Following Gynecological Malignancy: Time for Action! Climacteric. 2016;19:1–2.
[31] Wolf M, Kiesel L. Menopausale Hormonersatztherapie und gynäkologische Tumorerkrankungen. Gynäkologische Endokrinologie. 2011;9:161–164.
[32] Ortmann O, Emons G, Tempfer C. S3-Leitlinie: Hormonersatztherapie und Krebsrisiko. Gynäkologische Endokrinologie. 2020;18:20–25.
[33] Deli T, Orosz M, Jakab A. Hormone Replacement Therapy in Cancer Survivors - Review of the Literature. Pathol Oncol Res. 2019;.
[34] Kapoor E, Benrubi D, Faubion SS. Menopausal Hormone Therapy in Gynecologic Cancer Survivors: A Review of the Evidence and Practice Recommendations. Clin Obstet Gynecol. 2018;61:488–495.
[35] Edey KA, Rundle S, Hickey M. Hormone Replacement Therapy for Women Previously Treated for Endometrial Cancer. Cochrane Database Syst Rev. 2018;5:CD008830.
[36] Angioli R, Luvero D, Armento G, et al. Hormone Replacement Therapy in Cancer Survivors: Utopia? Crit Rev Oncol Hematol. 2018;124:51–60.
[37] O'Donnell RL, Clement KM, Edmondson RJ. Hormone Replacement Therapy after Treatment for a Gynaecological Malignancy. Curr Opin Obstet Gynecol. 2016;28:32–41.
[38] Biglia N, Bounous VE, Sgro LG, et al. Treatment of Climacteric Symptoms in Survivors of Gynaecological Cancer. Maturitas. 2015;82:296–298.
[39] Shim SH, Lee SJ, Kim SN. Effects of Hormone Replacement Therapy on the Rate of Recurrence in Endometrial Cancer Survivors: A Meta-Analysis. Eur J Cancer. 2014;50:1628–1637.
[40] Guidozzi F. Estrogen Therapy in Gynecological Cancer Survivors. Climacteric. 2013;16:611–617.
[41] Witczak K, Sajdak S, Kojs Z. Hormone Replacement Therapy in Gynecologic Oncology. Curr Gynecol Oncol. 2013;11:62–73.
[42] Manley K, Edey K, Braybrooke J, et al. Hormone Replacement Therapy after Endometrial Cancer. Menopause Int. 2012;18:134–138.
[43] Biliatis I, Thomakos N, Rodolakis A, et al. Safety of Hormone Replacement Therapy in Gynaecological Cancer Survivors. J Obstet Gynaecol. 2012;32:321–325.
[44] Tangjitgamol S, Manusirivithaya S, Hanprasertpong J, et al. Hormone Replacement Therapy after Treatment of Endometrial Cancer. Gynecol Obstet Invest. 2008;65:35–38.
[45] Biglia N, Mariani L, Marenco D, et al. Hormonal Replacement Therapy after Gynaecological Cancer. Gynakol Geburtshilfliche Rundsch. 2006;46:191–196.
[46] Schaudig K, Schwenkhagen A. Möglichkeiten der hormonellen Substitution bei gynäkologischen Malignomen. Gynakol Endokrinol. 2005;3:97–106.
[47] Biglia N, Gadducci A, Ponzone R, et al. Hormone Replacement Therapy in Cancer Survivors. Maturitas. 2004;48:333–346.
[48] Mueck AO, Seeger H. Hormone Therapy after Endometrial Cancer. Endocr Relat Cancer. 2004;11:305–314.
[49] Lin K, Runowicz CD. Hormone Replacement in the Patient with Uterine Cancer. In Coukos G, Rubin SC, editors, Cancer of the Uterus, 495–506. CRC Press. 2004;.
[50] Schindler AE. Hormone Replacement Therapy (HRT) in Women after Genital Cancer. Maturitas. 2002;41 Suppl 1:S105–111.
[51] Basil JB, Mutch DG. Role of Hormone Replacement Therapy in Cancer Survivors. Clin Obstet Gynecol. 2001;44:464–477.
[52] Lin K, Runowicz CD. The Wisdom of Hormone-Replacement Therapy in Survivors of Ovarian and Endometrial Cancer. Surg Clin North Am. 2001;81:987–993.
[53] Committee on Gynecologic Practice. ACOG Committee Opinion. Hormone Replacement Therapy in Women Treated for Endometrial Cancer. Number 234, May 2000 (Replaces Number 126, August 1993). Int J Gynaecol Obstet. 2001;73:283–284.
[54] Mulder JE. Benefits and Risks of Hormone Replacement Therapy in Young Adult Cancer Survivors with Gonadal Failure. Med Pediatr Oncol. 1999;33:46–52.
[55] Burger CW, van Leeuwen FE, Scheele F, et al. Hormone Replacement Therapy in Women Treated for Gynaecological Malignancy. Maturitas. 1999;32:69–76.
[56] Lentz SS. Endocrine Therapy of Endometrial Cancer. In Foon KA, Muss HB, Rosen ST, editors, Biological and Hormonal Therapies of Cancer, volume 94 of Cancer Treatment and Research, 88–106. Springer US, Boston, MA. 1998;.
[57] Wren BG. Hormone Therapy Following Breast and Uterine Cancer. Baillieres Clin Endocrinol Metab. 1993; 7:225–242.
[58] Hutchinson-Williams KA, Gutmann JN. Estrogen Replacement Therapy (ERT) in High-Risk Cancer Patients. Yale J Biol Med. 1991 Nov-Dec;64:607–626.
[59] Bock K, Hadji P, Schulz KD, et al. Therapiekonzepte bei klimakterischen Beschwerden für onkologische Patientinnen. Gynäkologe. 2003;36:479–486.
[60] Minig L, Franchi D, Valero de Bernabé J, et al. Controversies of the Hormonal Conservative Treatment of Endometrial Cancer. Gynecol Obstet Invest. 2013;75:145–151.
[61] Di Donato V, Palaia I, D'Aniello D, et al. Does Hormone Replacement Therapy Impact the Prognosis in Endometrial Cancer Survivors? A Systematic Review. Oncology. 2020;98:195–201.
[62] Brzozowska M, Lewinski A. Hormonal Replacement Therapy in Women with a History of Internal Genital Organ Malignancy. Prz Menopauzalny. 2021;20:34–39.
[63] Wren BG. Hormonal Therapy Following Female Genital Tract Cancer. Int J Gynecol Cancer. 1994; 4:217–224.
[64] Sommeijer DW, Sjoquist KM, Friedlander M. Hormonal Treatment in Recurrent and Metastatic Gynaecological Cancers: A Review of the Current Literature. Curr Oncol Rep. 2013;15:541–548.
[65] Warren MP, Halpert S. Hormone Replacement Therapy: Controversies, Pros and Cons. Best Pract Res Clin Endocrinol Metab. 2004;18:317–332.
[66] Killackey M. Hormone Replacement Therapy after a Cancer Diagnosis. Primary Care Update for OB/GYNS. 2002;9:85–89.
[67] Sourouni M, Kiesel L. Hormone Replacement Therapy After Gynaecological Malignancies: A Review Article. Geburtshilfe Frauenheilkd. 2021;81:549–554.
[68] Gambacciani M, Monteleone P, Sacco A, et al. Hormone Replacement Therapy and Endometrial, Ovarian and Colorectal Cancer. Best Practice & Research Clinical Endocrinology & Metabolism. 2003;17:139–147.
[69] de Araújo NLC, Athanazio DA. [Hormone replacement therapy and endometrial cancer]. Cad Saude Publica. 2007;23:2613–2622.
[70] Krauss K, Rossmanith W. Hormone Replacement Therapy and Gynecologic Cancer. Oncol Res Treat. 1997;20:448–454.
[71] Richardson A, Ayres J, Cust M, et al. Hormone Replacement Therapy Following Treatment of Gynaecological Malignancies. Obstet Gynecol. 2019;21:291–298.
[72] Ibeanu O, Modesitt SC, Ducie J, et al. Hormone Replacement Therapy in Gynecologic Cancer Survivors: Why Not? Gynecol Oncol. 2011;122:447–454.
[73] Pecorelli S, Fallo L. Hormone Replacement Therapy in Gynecological Cancer Survivors. Crit Rev Oncol Hematol. 1998;27:1–10.
[74] Loizzi V, Cormio G, Vicino M, et al. Hormone Replacement Therapy on Ovarian and Uterine Cancer Risk and Cancer Survivors: How Shall We Do No Harm? Int J Gynecol Cancer. 2005 May-Jun;15:420–425.
[75] Lattrich C, Schüler S, Mögele M, et al. Hormonsubstitution bei gynäkologischen Malignomen. Gynäkologe. 2012;45:618–622.

[76] Sinno AK, Pinkerton J, Febbraro T, et al. Hormone Therapy (HT) in Women with Gynecologic Cancers and in Women at High Risk for Developing a Gynecologic Cancer: A Society of Gynecologic Oncology (SGO) Clinical Practice Statement: This Practice Statement Has Been Endorsed by The North American Menopause Society. Gynecol Oncol. 2020;157:303–306.
[77] Sismondi P, Biglia N, Giai M, et al. HRT, Breast and Endometrial Cancers: Strategies and Intervention Options. Maturitas. 1999;32:131–139.
[78] Purohit P, Sassarini J, Lumsden MA. Management of Induced Menopause in Gynaecological Cancers and Their Challenges. Curr Obstet Gynecol Rep. 2019;8:94–102.
[79] Brennan A, Brennan D, Rees M, et al. Management of Menopausal Symptoms and Ovarian Function Preservation in Women with Gynecological Cancer. Int J Gynecol Cancer. 2021;31:352–359.
[80] Marino JL, McNamara HC, Hickey M. Managing Menopausal Symptoms after Cancer: An Evidence-Based Approach for Primary Care. Med J Aust. 2018;208:127–132.
[81] Fenton CL, McGauran MJ, Richards AM. Menopausal Hormonal Therapy in Gynaecological Cancers: An Evidence-Based Approach for Clinical Management. Aust N Z J Obstet Gynaecol. 2020;60:942–945.
[82] Kuhle CL, Kapoor E, Sood R, et al. Menopausal Hormone Therapy in Cancer Survivors: A Narrative Review of the Literature. Maturitas. 2016;92:86–96.
[83] Ahn HJ, Hwang KR. Menopausal hormone therapy in the cancer survivors. J Korean Med Assoc. 2019; 62:160.
[84] Kushtagi P, Narayanan A. Ovarian Hormone Replacement Therapy in Gynecological Cancer Survivors. Indian J Gynecol Oncolog. 2016;14:33.
[85] Jacob SS, Somashekhar SP, Jacob SS. Role of Hormone Replacement Therapy (HRT) in Gynecological Cancers: Endocrinologist's Perspective. Indian J Gynecol Oncolog. 2016;14:43.
[86] Buchholz S, Ortmann O. Behandlung von klimakterischen Symptomen bei Frauen mit gynäkologischen Malignomen und Mammakarzinom. Gynäkologe. 2013;46:160–164.
[87] Peters ITA, Brownfoot FC, Trimbos JB, et al. What Is the Place of Hormone Replacement Therapy in Ovarian, Endometrial, and Breast Cancer? In Ledermann JA, Creutzberg CL, Quinn MA, editors, Controversies in the Management of Gynecological Cancers, 237–246. Springer London, London. 2014;.
[88] McDonnell BA, Twiggs LB. Hormone Replacement Therapy in Endometrial Cancer Survivors: New Perspectives after the Heart and Estrogen Progestin Replacement Study and the Women's Health Initiative. J Low Genit Tract Dis. 2006;10:92–101.
[89] Del Carmen MG, Rice LW. Management of Menopausal Symptoms in Women with Gynecologic Cancers. Gynecol Oncol. 2017;146:427–435.
